# Intravitreal ranibizumab or conbercept for retinal arterial macroaneurysm: a case series

**DOI:** 10.1186/s12886-019-1035-z

**Published:** 2019-01-15

**Authors:** Zhongjing Lin, Qiwei Hu, Yanlin Wu, Jianmin Xu, Qiong Zhang

**Affiliations:** Department of Ophthalmology, Ruijin Hospital Affiliated Medical School, Shanghai Jiaotong University, 197 Ruijin Er Road, Shanghai, 200025 China

**Keywords:** Retinal arterial macroaneurysm, Ranibizumab, Conbercept

## Abstract

**Background:**

There is no consensus for the standard treatment of retinal arterial macroaneurysm (RAM). Intravitreal anti-vascular endothelium growth factor (anti-VEGF) is an alternative treatment option for RAM. The purpose of this study is to describe the clinical efficacy of intravitreal ranibizumab or intravitreal conbercept for retinal arterial macroaneurysm.

**Case presentation:**

Three cases that presented with symptomatic RAM were treated with intravitreal anti-VEGF agents. Two eyes received two intravitreal ranibizumab injections with a time interval of one month and completed a one-year follow-up, while one eye only received one intravitreal conbercept injection and was followed up for six months. Both the retinal thickness and the visual acuity were significantly improved at the final clinic visit. The macular hemorrhage and edema were resolved. There were no ocular or systemic side effects.

**Conclusions:**

Intravitreal ranibizumab or conbercept might be used as a therapeutic option for symptomatic retinal arterial macroaneurysm patients. Anti-VEGF therapy should be further investigated in a larger series with longer follow-up for this disease profile.

## Background

Retinal arterial macroaneurysm (RAM) was first reviewed by Robertson in 1973 [1]. It usually occurs within the first three orders of arterial bifurcation with the morphology of fusiform or saccular dilatations [2]. The most common risk factors include aging, systemic vascular pathology (hypertension, dyslipidemia and atherosclerosis) and the female gender [3]. The majority of RAMs will spontaneously resolve, and even with complications occurred such as submacular hemorrhage and edema involving the fovea, up to 37% of patients will maintain best corrected visual acuity (BCVA) better than 20/40 without intervention [4]. However, approximately one-third of symptomatic RAMs need to be treated due to the visual impairment caused by the retinal edema and hemorrhage [3]. There are several treatment options with various outcomes [5–7], yet no consensus has been reached on the most effective. Recent studies have revealed that intravitreal injection with anti-vascular endothelium growth factor (anti-VEGF) drugs could provide encouraging outcomes [7–10]. In our paper, we presented the clinical features and outcomes in three cases of symptomatic RAMs treated with anti-VEGF agents.

## Case presentation

Table 1 summarizes the characteristics of the three patients and their treatment results. We performed a step-by-step workup including patient history, clinical ophthalmological assessment (BCVA, slit-lamp biomicroscopy, fundus examination, intraocular pressure), optical coherence tomography (OCT), fundus fluorescein angiography (FA), and indocyanine green angiography (ICGA), if applicable. OCT angiograms were obtained in case 2 and case 3, since the Cirrus OCT with AngioPlex™ was put into service. None of the patients had evidence of systemic disease except for a documented history of hypertension. Since anti-VEGF agents were off-label injections in this situation, after explaining the possible advantages and outcomes of currently available anti-VEGF agents for the management of RAM, the patients made their own decision whether to use ranibizumab or conbercept. No complications, such as endophthalmitis, traumatic lens injury or retinal detachment were observed with the intravitreal injection.

**Table 1:** The Characteristics of three patients with retinal arterial macroaneurysm

Case	Age (years)	Sex	Eye	Medical history	Lesion location	Fovea	Anti-VEGF injection	Follow-up (months)	BCVA		CMT(μm)	
									baseline	final	baseline	final
1	69	female	left	HT	supra-temporal	IRH, SRD	ranibizumab # 2	12	20/70	20/30	392	205
2	76	female	right	HT	infra-temporal	IRH, SRD	ranibizumab #2	12	20/200	20/50	465	183
3	62	female	left	HT	infra-temporal	PRH	conbercept #1	6	CF	20/40	799	313

*HT* hypertension, *IRH* intra-retinal hemorrhage, *PRH* pre-retinal hemorrhage, *SRD* serous retinal detachment, *BCVA* best-corrected visual acuity, *CF* counting fingers, *CMT* central macular thickness

### Case 1

**Fig. 1:**
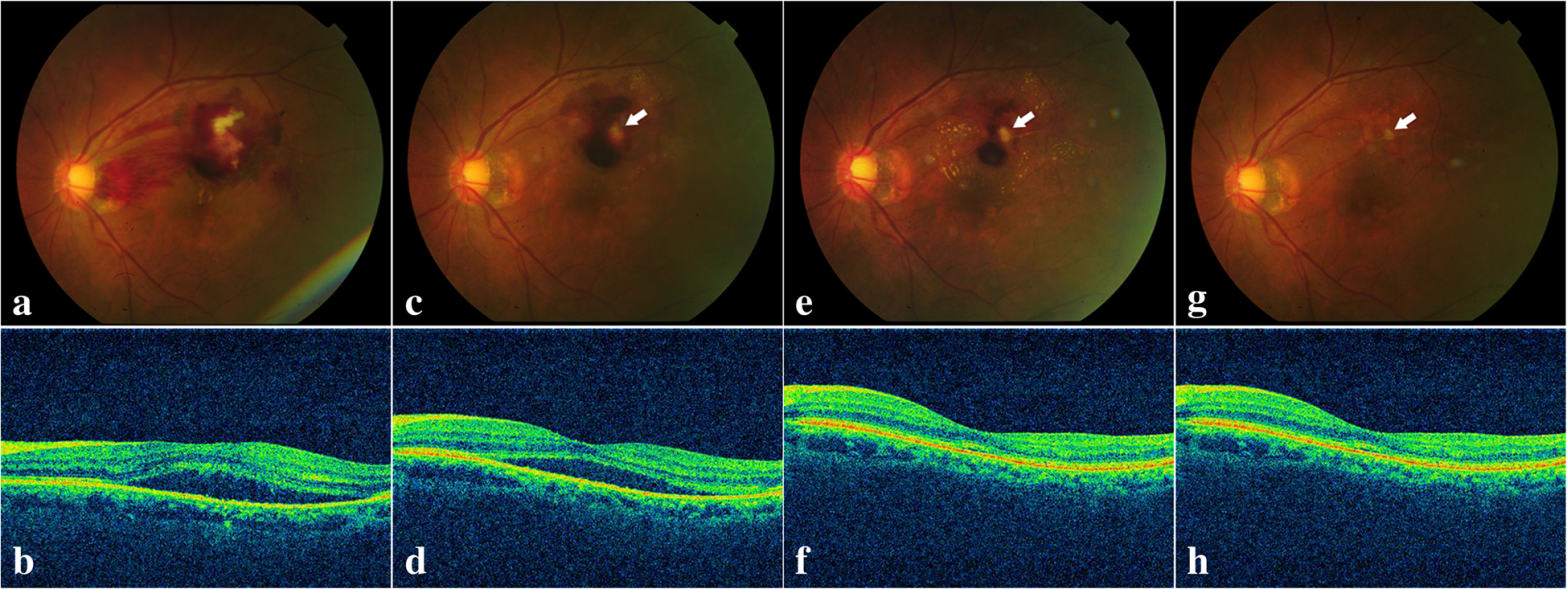
Multimodal imaging of the affected eye in case 1. **a**. Fundus photography showing the supra-temporal hemorrhage and white lesion above the macula at the baseline visit. **b**. Corresponding OCT scan through the fovea showing SRD at the baseline visit. **c**. One month after the first injection, fundus photography showing the diminished hemorrhage site and dominant white fibrosis (arrow). **d**. Corresponding OCT scan through the fovea showing SRD. **e**. One month after the second injection, fundus photography showing the further diminished hemorrhage site and dominant white fibrosis (arrow). **f**. Corresponding OCT scan through the fovea showing total resolution of SRD. **g**. Fundus photography showing no hemorrhage and only white fibrosis left (arrow) at the one-year follow up. **h**. Corresponding OCT scan through the fovea showing normal appearance of the macular

A 69-year-old female presented with vision reduction and metamorphopsia in her left eye for at least 3 weeks. Her baseline BCVA was 20/70. Fundus photography (Fig. 1a) showed the intraretinal hemorrhage with a white lesion above the macula. Since the patient was allergic to the fluorescein sodium, there was no FA result. ICGA was not performed due to the short of the contrast agent. OCT scans through the fovea showed serous retinal detachment (SRD) (Fig. 1b). With the diagnosis of a possible ruptured RAM, the patient received the first intravitreal ranibizumab injection. Four weeks after the first injection, the fundus examination (Fig. 1c) showed that the bleeding diminished and the white lesion (fibrosis) was more dominant than before. The BCVA did not change. Due to the sustained SRD in the macula (Fig. 1d), a second intravitreal ranibizumab injection was administered at this visit. Subsequently, one month later, her visual acuity had improved to 20/40. Fundus photography (Fig. 1e) showed further resolution of the fundus hemorrhage, and only white fibrosis (RAM atrophy) in the superior temporal artery remained. The OCT scan showed the total resolution of SRD (Fig. 1f). At the one-year follow-up, her BCVA was maintained at 20/30. Fundus examination (Fig. 1g) confirmed the completed absorption of the hemorrhage, and OCT scans showed a well-preserved macular appearance (Fig. 1h). Unfortunately, the patient sustained a cerebral infarction after one year, and the remaining follow-up was terminated.

### Case 2

**Fig. 2:**
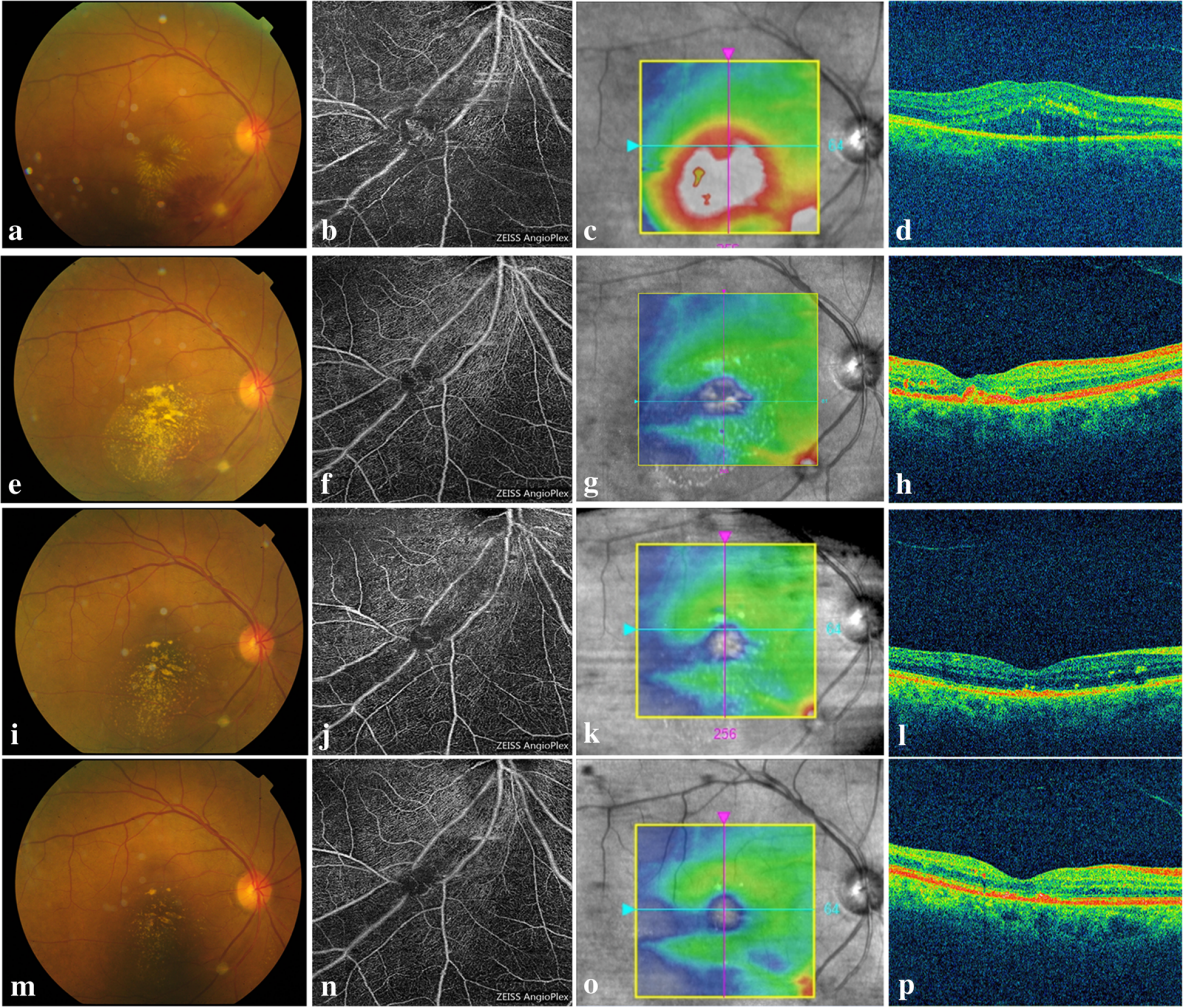
Multimodal imaging of the affected eye in case 2. **a**. Fundus photography showing infra-temporal RAM with surrounding stellate-shaped exudates involving the fovea at the baseline visit. **b**. OCT angiography showing the heliciform capillary mass in the RAM in the superficial layer at the baseline visit. **c**. Corresponding image showing the OCT B-scan lines. **d**. OCT scan showing SRD in the macular area at the baseline visit. **e**. Fundus photography showing the absorption of the hemorrhage and the remaining white fibrosis and hard exudates involving the fovea one month after the first injection. **f**. OCT angiography showing the regression of the heliciform capillary mass in the RAM in the superficial layer one month after the first injection. **g**. Corresponding image showing the OCT B-scan lines. **h**. OCT scan showing disappeared SRD in the macular area one month after the first injection. **i**. Fundus photography showing resolved hard exudates one month after the second injection. **j**. OCT angiography showing the further regression of the heliciform capillary mass in the RAM in the superficial layer one month after the second injection. **k**. Corresponding image showing the OCT B-scan lines. **l**. CT scan through the fovea showing total resolution of SRD one month after the second injection. **m**. Fundus photography showing no hemorrhage and only white fibrosis (arrow) in the inferior-temporal artery at the one-year follow up. **n**. OCT angiography showing the absence of the capillary mass at the one-year follow up. **o**. Corresponding image showing the OCT B-scan lines. **p**. OCT scan showing normal macular appearance without SRD at the one-year follow up

**Fig. 3:**
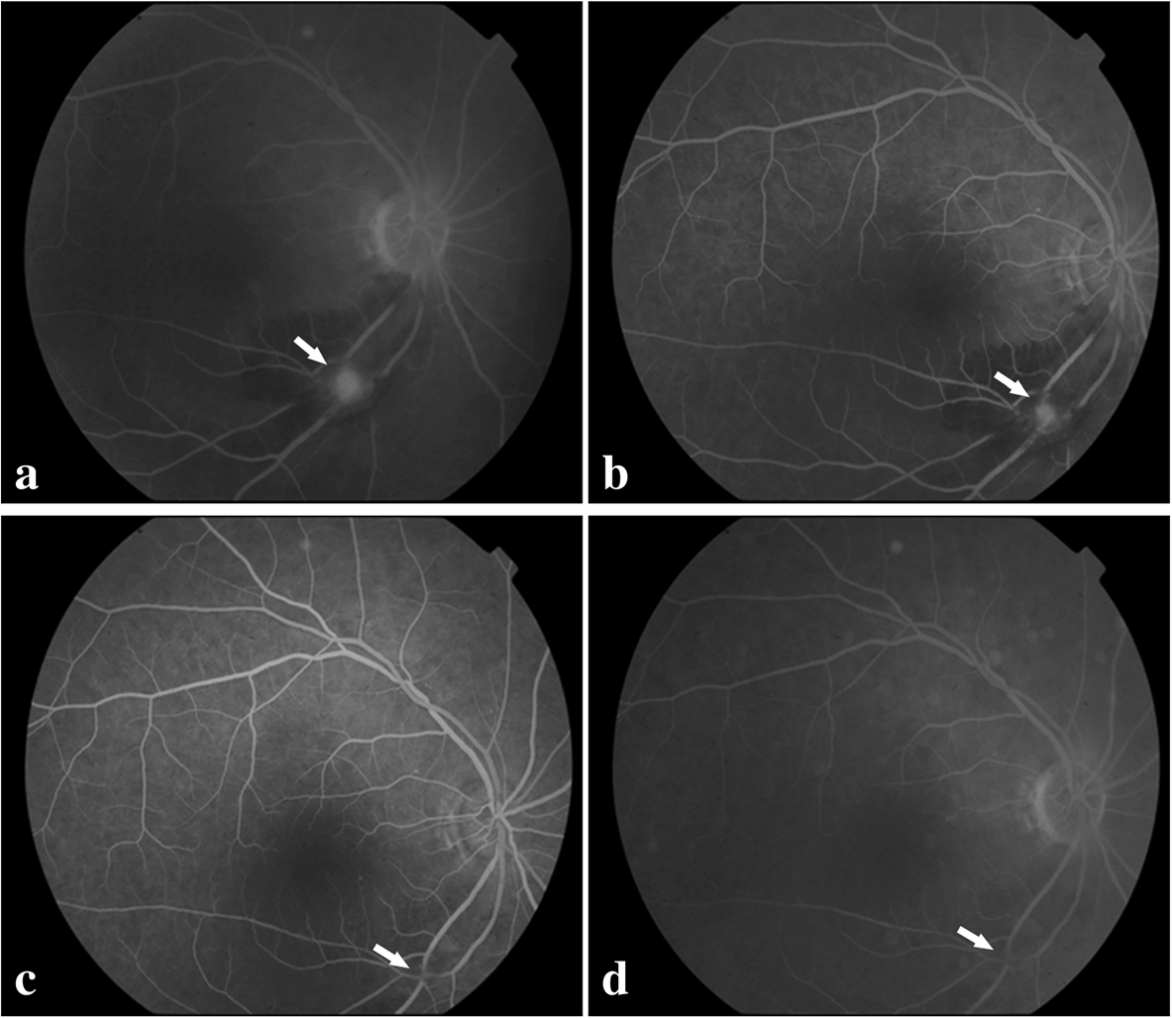
Multimodal imaging of the affected eye in case 2. **a**. FA image at the early phase showing fluorescein filling of the macroaneurysm (arrow) at the baseline visit. **b**. FA image at the late phase showing mild leakage (arrow) at the baseline visit. **c**. FA image at the early phase showing resolution of the macroaneurysm leaving focal perivascular fibrosis (arrow) at the one-year follow up. **d**. FA image at the late phase showing resolution of the macroaneurysm leaving focal perivascular fibrosis (arrow) at the one-year follow up

A 76-year-old female developed a deterioration of visual acuity in her right eye for approximately one month. The BCVA was 20/200 in the right eye. Dilated fundus examination (Fig. 2a) and FA (Fig. 3a-b) revealed infra-temporal RAM, with surrounding stellate-shaped exudates involving the fovea. The OCT angiogram further confirmed the RAMs (Fig. 2b). And a heliciform capillary mass in the RAMs was observed in the superficial layer (segmented with an inner boundary at 3 μm beneath the internal limiting membrane and outer boundary at 15 μm beneath the inner plexiform layer). SRD was also observed in the OCT scan through the fovea (Fig. 2c-d). Her vision was improved to 20/70, accompanied by the increased hard exudate around the fovea (Fig. 2e) and the resolution of SRD one month after the first intravitreal injection of 0.5 mg of ranibizumab (Fig. 2f-h). Considering the therapy regimen used for neovascular age-related macular degeneration (AMD), we continued treatment with a second intravitreal injection (ranibizumab). One month after the second injection, the hard exudate diminished (Fig. 2i-l) and the BCVA improved to 20/50. At the one-year follow-up, the final fundus examination (Fig. 2m) and FA (Fig. 3c-d) confirmed the complete absorption of the hemorrhage and the atrophy of RAM. The OCT angiography showed that the capillary mass in the RAM disappeared (Fig. 2n). The macular anatomy maintained a normal appearance without SRD at the final visit (Fig. 2o-p).

### Case 3

**Fig. 4:**
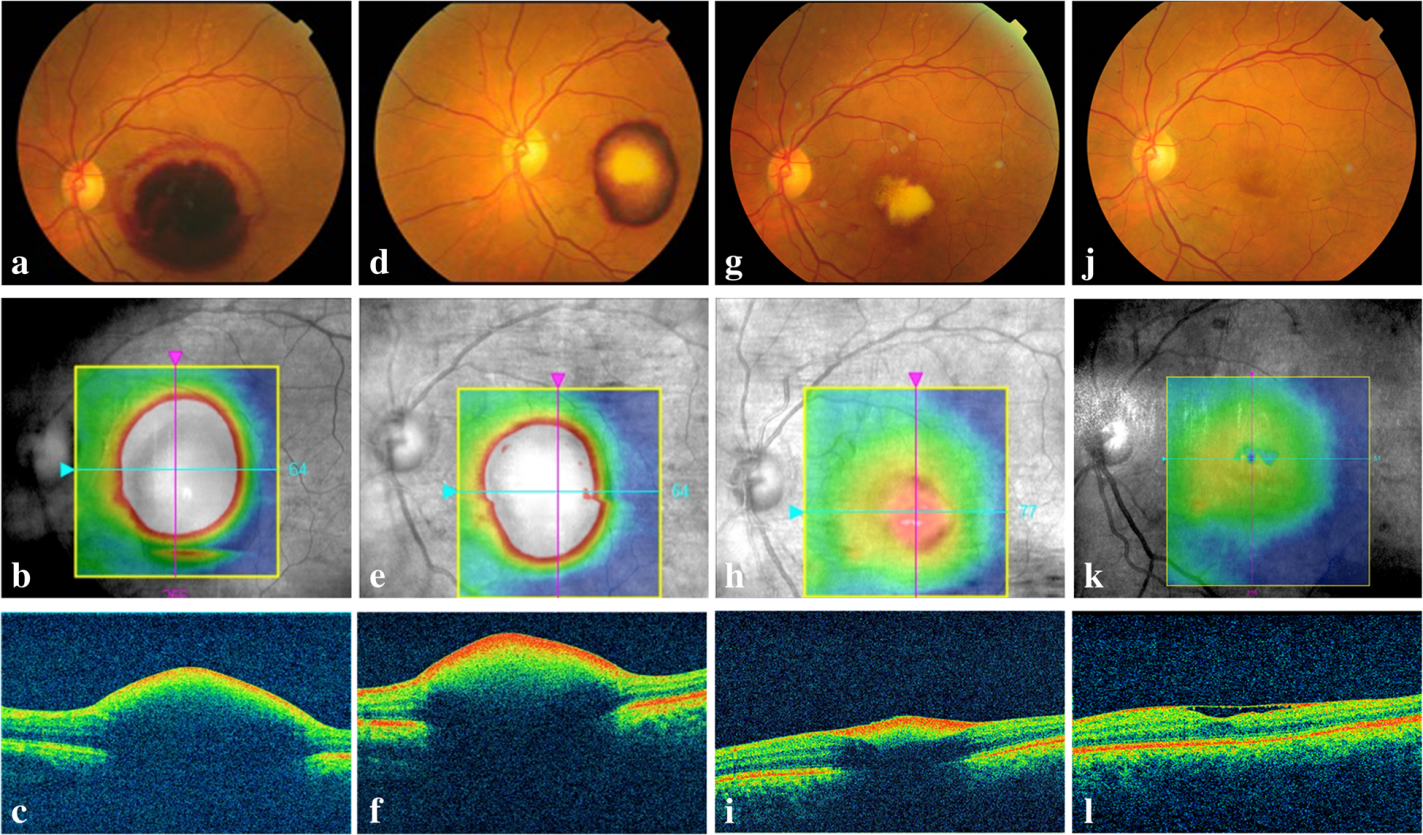
Multimodal imaging of the affected eye in case 3. **a**. Color photography at baseline showing the macular hemorrhage involving the posterior pole. **b**. Corresponding image showing the OCT B-scan lines. **c**. OCT through the fovea showing the preretinal hemorrhage and increased CMT at baseline visit. **d**. Color photography showing the partial absorption of the hemorrhage one month after the first injection. **e**. Corresponding image showing the OCT B-scan lines. **f**. OCT scan showing the decreasing CMT one month after the first injection. **g**. Color photography showing the further absorption of the hemorrhage at the 3-month follow-up. **h**. Corresponding image showing the OCT B-scan lines. **i**. OCT scan showing further decreased CMT at the 3-month follow-up. **j**. Color photography at the 6-month follow-up showing almost no hemorrhage and a red lesion at the inferior temporal artery. **k**. Corresponding image showing the OCT B-scan lines. **l**. OCT scan at 6-month follow-up showing almost no SRD

**Fig. 5:**
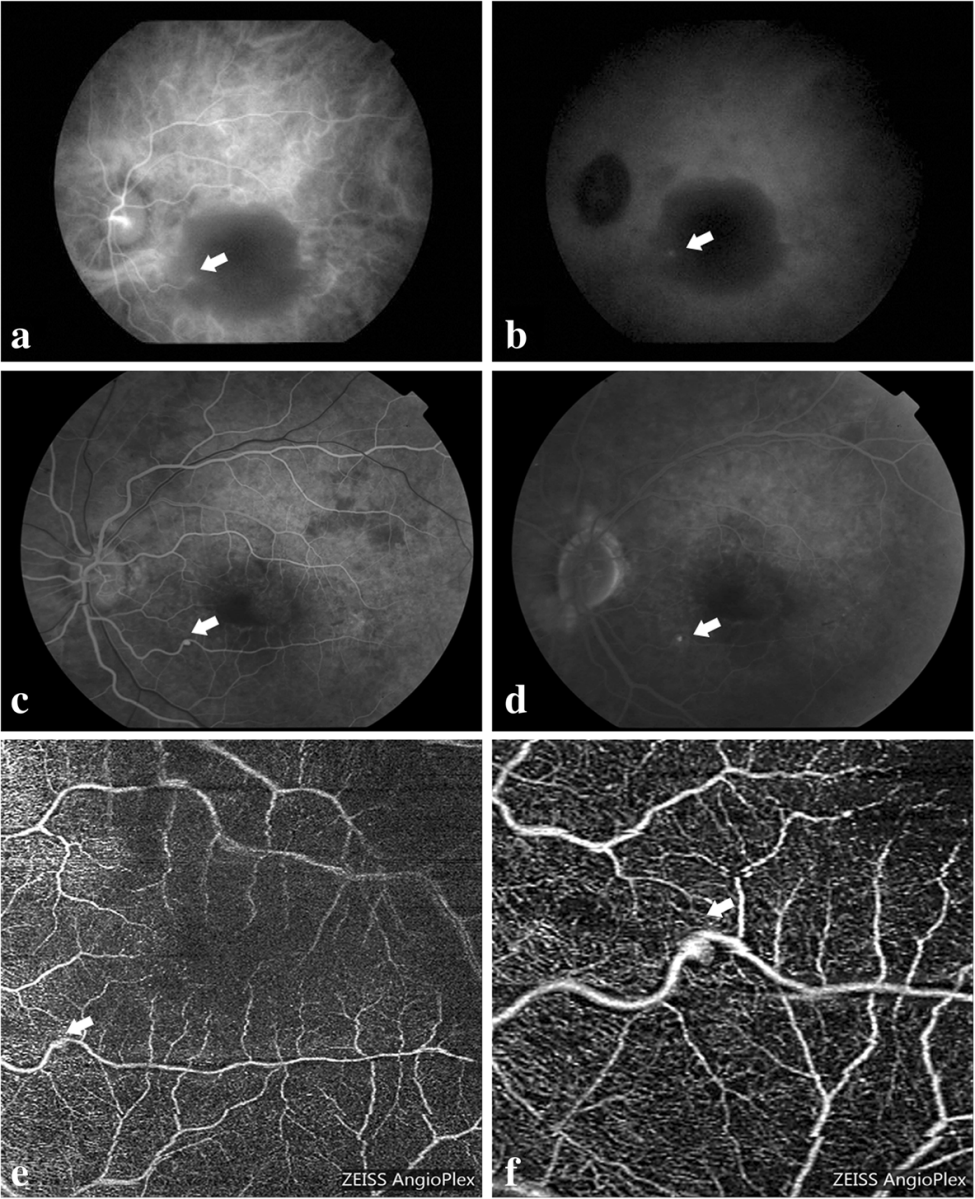
Multimodal imaging of the affected eye in case 3. **a**. ICGA at the early phase showing hyperfluoresence at the infratemperal area of the edge of the dark area at the baseline visit. **b**. ICGA at the late phase showing the continuing lighting in the same area at the baseline visit. **c**. FA at the early phase showing fluorescein filling of the macroaneurysm at the 6-month follow-up. **d**. FA at the late phase showing not faded fluorescein filling of the macroaneurysm at the 6-month follow-up. **e**. OCT-A 6 mm × 6 mm superficial slab revealed the RAM at the 6-month follow-up. **f**. OCT-A 3 mm × 3 mm superficial slab clearly delineated the RAM at the 6-month follow-up. White arrow indicated the macroaneurysm

A 62-year-old female experienced sudden visual loss in her left eye for approximately two weeks. The BCVA was limited to counting fingers in the left eye. Dilated fundus examination (Fig. 4a) showed preretinal hemorrhage in the macula area. OCT (Fig. 4b-c) scanning of the fovea showed markedly increased retinal thickness. Considering the dense hemorrhage, we only conducted an ICGA examination. The results showed hyperfluoresence at the inferior temporal area of the edge of the dark area (Fig. 5a-b). A possible diagnosis of RAM was made. After explaining the possible advantages and outcomes of anti-VEGF therapies, the patient chose intravitreal conbercept (0.5 mg) injection for economic reasons. One month after the first injection, the BCVA did not change. However, the color fundus image (Fig. 4d) showed the partial absorption of the hemorrhage and a decrease in central macular thickness (673 μm) (Fig. 4e-f). The patient refused another intravitreal injection for economic reasons. Two months later, the BVCA improved to 20/400, and the corresponding examinations showed satisfactory results (Fig. 4g-i). At the final clinic visit, six months after the initial visit, her BCVA greatly improved to 20/40, and the ocular findings suggested that the hemorrhage was well absorbed (Fig. 4j-l). The FA results showed that the macroaneurysm in the inferior temporal artery was fluorescence filled at the early phase (Fig. 5c) and it did not fade at the late phase (Fig. 5d). The OCT-A superficial slab also clearly delineated the site of RAM, which was consistent with the FA results (Fig. 5e-f).

## Discussion

RAMs are rare, acquired, localized dilation of retinal arterial branches, possibly resulting in macular edema, serous retinal detachment with subretinal fluid, and intraretinal lipid accumulation [11]. The abrupt rupture of RAMs may cause subretinal, intraretinal, preretinal or vitreous hemorrhage. Since the majority of RAM patients have a benign course, asymptomatic or spontaneous resolution, close follow-up is a reasonable treatment option. However, it is challenging to identify patients who regress spontaneously. In symptomatic RAM cases, if the macular is not involved, observation is still considered to be the preferred treatment. When the vision decreases and the macular is involved in hemorrhage or exudation, patients can still receive close follow-up in the early stage due to the high possibility of spontaneous resolution. Early intervention depends on the individual’s decision and their visual needs. However, chronic and long-lasting hemorrhage or exudation, especially in the subretinal layer, could cause the progressive damage of photoreceptors, leading to permanent visual disorders. Nonresolving cases may be complicated by a rupture of the RAM wall and significant preretinal hemorrhage, which could result in visual deterioration or surgical intervention. Thus, early intervention is recommended, especially in cases with foveal involvement. The most common treatment is direct argon laser photocoagulation at the site of RAM, but with several complications, such as possible retinal traction, an increase in retinal exudation, and enlargement of the laser scar, which limit the use in the clinical practice to some extent [11, 12]. Other therapeutic options include pneumatic displacement of tissue by a plasminogen activator, surgical removal of the associated hemorrhage with vitrectomy, and photodisruption of the internal limiting membrane or the posterior hyaloid to release the hemorrhage [11]. However, there has been no established treatment protocol available.

Recently, intravitreal injections of anti-VEGF agents have been advocated for the management of RAM patients with the macular hemorrhage or edema. Chanana and Azad [13] published the first case report in 2009, and subsequent case reports have shown encouraging results [6–10]. Intravitreal injections of bevacizumab [8, 9, 13], ranibizumab [14, 15], or aflibercept [10, 14] could lead to rapid and complete resolution of macular edema and hemorrhage, as well as significant visual recovery. However, the mechanism of the therapy remains unclear. Focal embolic damage to arterial walls is considered to cause RAMs, thus VEGF might be upregulated secondary to localized ischemia and hypoxia, which result in increased vascular permeability by stimulating nitrogen oxide production in the endothelium, as well as the activation of coagulation cascades. Anti-VEGF agents may cause vasoconstriction and reduce nitrogen oxide production, which provide a beneficial role in the resolution of macular edema. Besides, these agents may improve the balance between coagulation and fibrinolysis, which facilitates the dissipation of macular hemorrhage [8, 11, 14, 16, 17]. In our paper, case 1 and case 2 received intravitreal ranibizumab injections and still achieved satisfactory anatomical and visual results at the one-year follow-up. The visual function was improved rapidly after the first injection. Although the edema resolved, the hard exudates after the first injection were more prominent in case 2, so the patients received another treatment. Exudates may be related to increased phagocytotic activity in the retinal tissue. In addition, in case 3, we also described the treatment of a ruptured RAM complicated by preretinal hemorrhage who was treated with intravitreal conbercept injection.

Conbercept is a fusion protein that has the binding domains of all isoforms VEGF-A, VEGF-B, VEGF-C and placental growth factor [18–20]. It has a similar structure to aflibercept with the addition of the binding domain of VEGF-C. Prior studies have reported that conbercept has promising effects on the neovascular AMD [18, 21], diabetic retinopathy [19, 22], polypoidal choroidal vasculopathy [23], retinopathy of prematurity [24], and macular edema secondary to branch retinal vein occlusion [25]. In case 3, we used ICGA to assist with the diagnosis because of the dense hemorrhage. After treatment with conbercept, the macular hemorrhage and edema were regressed; thus, BCVA was improved. The therapy was well tolerated without any adverse events. Considering the therapy regimen used for AMD, we recommended a second intravitreal injection. However, the patient refused for economic reasons. Nevertheless, we were surprised to find improved BCVA and further diminished hemorrhage in the follow-up periods. After the hemorrhage and edema diminished, we observed the infratemporal RAM and FA results presented positive results. This outcome may imply that we could follow up with observation one month after the first injection instead of administering the second injection. However, further investigation in a larger series with longer follow-up is needed to verify the clinical efficacy. To the best of our knowledge, this is the first report of intravitreal conbercept injection for RAMs.

## Conclusion

Intravitreal ranibizumab or conbercept might be used as a therapeutic option for symptomatic retinal arterial macroaneurysm patients. Anti-VEGF therapy should be further investigated in a larger series with longer follow-up for this disease profile.

## Abbreviations

AMD: Age-related macular degeneration
Anti-VEGF: Anti-vascular endothelium growth factor
BCVA: Best corrected visual acuity
FA: Fluorescein angiography
ICGA: Indocyanine green angiography
OCT: Optical coherence tomography
RAM: Retinal arterial macroaneurysm
SRD: Serous retinal detachment
